# Dynamics of Optically-Generated Carriers in Si (100) and Si (111) Substrate-Grown GaAs/AlGaAs Core-Shell Nanowires

**DOI:** 10.1186/s11671-015-1050-9

**Published:** 2015-08-21

**Authors:** Ramon delos Santos, Jasher John Ibañes, Maria Herminia Balgos, Rafael Jaculbia, Jessica Pauline Afalla, Michelle Bailon-Somintac, Elmer Estacio, Arnel Salvador, Armando Somintac, Christopher Que, Satoshi Tsuzuki, Kohji Yamamoto, Masahiko Tani

**Affiliations:** National Institute of Physics, University of the Philippines, Diliman, Quezon City, Philippines; Department of Physics, Ateneo de Manila University, Quezon City, Philippines; Department of Physics, De La Salle University, Manila, Philippines; Research Center for Development of Far-Infrared Region, University of Fukui, Fukui, Japan

**Keywords:** Optical properties of nanowires, Time-resolved spectroscopy, Infrared and Raman spectra of III-V semiconductors, Molecular beam epitaxy, Semiconducting gallium arsenide, Optical properties of semiconductors, 78.67.Uh, 78.47.D-, 78.30.Fs

## Abstract

GaAs/Al_0.1_Ga_0.9_As core-shell nanowires (CSNWs), with average lateral size of 125 nm, were grown on gold nanoparticle-activated Si (100) and Si (111) substrates via molecular beam epitaxy. Room temperature-photoluminescence (RT-PL) from the samples showed bulk-like GaAs and Al_0.1_Ga_0.9_As bandgap emission peaks at 1.43 and 1.56 eV, respectively. Higher PL emission intensity of the sample on Si (111) compared to that on Si (100) is attributed to uniform Al_0.1_Ga_0.9_As shell passivation of surface states on Si (111)-grown CSNWs. Carrier dynamics in two different temporal regimes were studied. In the sub-nanosecond time scale (300–500 ps), time-resolved radiative recombination efficiency of carriers was examined. In the 0–4 ps range, surface field-driven ballistic transport of carriers was probed in terms of the radiated terahertz (THz) waves. Time-resolved PL measurements at 300 K revealed that the carrier recombination lifetime of the GaAs core on Si (100)-grown CSNWs is 333 ps while that on Si (111)-grown sample is 500 ps. Ultrafast photoexcitation of GaAs core on the two samples generated a negligible difference in the intensity and bandwidth of emitted THz radiation. This result is ascribed to the fact that the deposited GaAs material on both substrates produced samples with comparable NW densities and similar GaAs core average diameter of about 75 nm. The samples’ difference in GaAs core’s carrier recombination lifetime did not influence THz emission since the two processes involve distinct mechanisms. The THz spectrum of CSNWs grown on Si (111) exhibited Fabry-Perot modes that originated from multiple reflections of THz waves within the Si substrate.

## Background

Semiconducting nanowire (NW)-based devices are considered to be versatile building blocks for optical and optoelectronic circuits at the nanoscale [1–7]. The compound semiconductor GaAs is one of the most viable choices for NW material due to the already existing technological platform. Epitaxial growth of GaAs NWs on Si substrate is of considerable interest because it has paved the way for the integration of III-V semiconductor nanoscale devices with well-established Si technology [8]. Nevertheless, the optical performance of bare GaAs NWs is relatively poor as a result of sensitivity to surface states that act as nonradiative carrier traps in the NWs [9, 10]. To surmount this problem, a higher bandgap material like AlGaAs can be grown as a passivation layer on the facets of GaAs NWs to form a structure called core-shell NWs (CSNWs). The higher bandgap material prevents interaction of the carriers in the GaAs core with the surface states [9, 10].

In this paper, the dynamics of optically-generated carriers in GaAs/AlGaAs CSNWs grown on Si (100) and Si (111) substrates were investigated. Room temperature-photoluminescence (RT-PL) spectroscopy was utilized to determine material composition of CSNWs and the quality of AlGaAs shell. Carrier dynamics in the sub-nanosecond time scale was examined via time-resolved PL (TRPL) spectroscopy while that in the 0–4 picoseconds (ps) regime was probed using terahertz-time domain spectroscopy (THz-TDS). In particular, TRPL was employed to measure GaAs core’s carrier recombination lifetime. On the other hand, the main purpose of THz-TDS measurement is to shed light on the drift of photoexcited carriers by the strong surface depletion field. Ultrafast optical excitation of GaAs generates current surge, which is associated with the drift of photoexcited carriers and is manifested in terms of a transient THz wave emission [11, 12]. The THz electric field amplitude is proportional to the time derivative of the surge current [13, 14]. Using THz-TDS measurements, GaAs core’s THz emission from samples on Si (100) and Si (111) were compared to that of a semi-insulating (SI)-GaAs (100) substrate.

## Methods

GaAs/AlGaAs CSNWs were synthesized using anodized aluminum oxide (AAO)-patterned gold (Au) nanoparticles on Si (100) and Si (111) substrates. A single nanoparticle array template was fabricated on these substrates to initiate the vapor-liquid-solid (VLS) growth of GaAs core. Samples were grown side-by-side in a Riber 32P molecular beam epitaxy (MBE) chamber to ensure similar growth conditions. The CSNW growth sequence started with the deposition of GaAs at 580 °C for 30 min to form the GaAs core, followed by AlGaAs deposition for 30 min on the surfaces of GaAs NWs to form the core-shell structure. A thin Si-doped GaAs cap layer was also grown over the core-shell to prevent oxidation of the AlGaAs shell. To approximate the core diameter of CSNW samples, uncoated GaAs NWs were grown using the same growth parameters.

For the investigation of surface morphology and steady-state optical properties of the as-grown CSNW samples, field emission scanning electron microscopy (FE-SEM) and PL spectroscopy analyses were performed, respectively. SEM images were obtained in a Philips XL 30 Field Emission Gun (FEG) system. PL measurements were done using a 488 nm continuous-wave Ar^+^ laser as an excitation source. The output signal was collected by a SPEX 500 M monochromator and detected by a Hamamatsu GaAs photomultiplier tube (PMT). TRPL experiments were carried out using a frequency doubled Ti/Sapphire femtosecond laser (400 nm) with 100 fs pulses at 80 MHz repetition rate. The resulting 800 nm excitation wavelength did not excite the AlGaAs shell. The GaAs PL signal was made available to a streak camera system consisting of Digikröm spectrometer with an Optronis streak camera attached to it.

THz emission of the CSNW samples and SI-GaAs (100) wafer was measured via a standard THz-TDS system in 45°-reflection excitation geometry mode. Ultrafast photoexcitation of GaAs material was performed using a mode-locked Ti/Sapphire laser as an excitation source, centered at 850 nm wavelength, emitting 100 fs pulses at a repetition rate of 80 MHz. The time domain plots of THz transients were detected by optically gated, low temperature-grown GaAs photoconductive antenna. The 850 nm excitation wavelength generated THz emission from the GaAs core only since the associated energy of the laser is not adequate to excite the AlGaAs shell.

## Results and Discussion

**Fig. 1:**
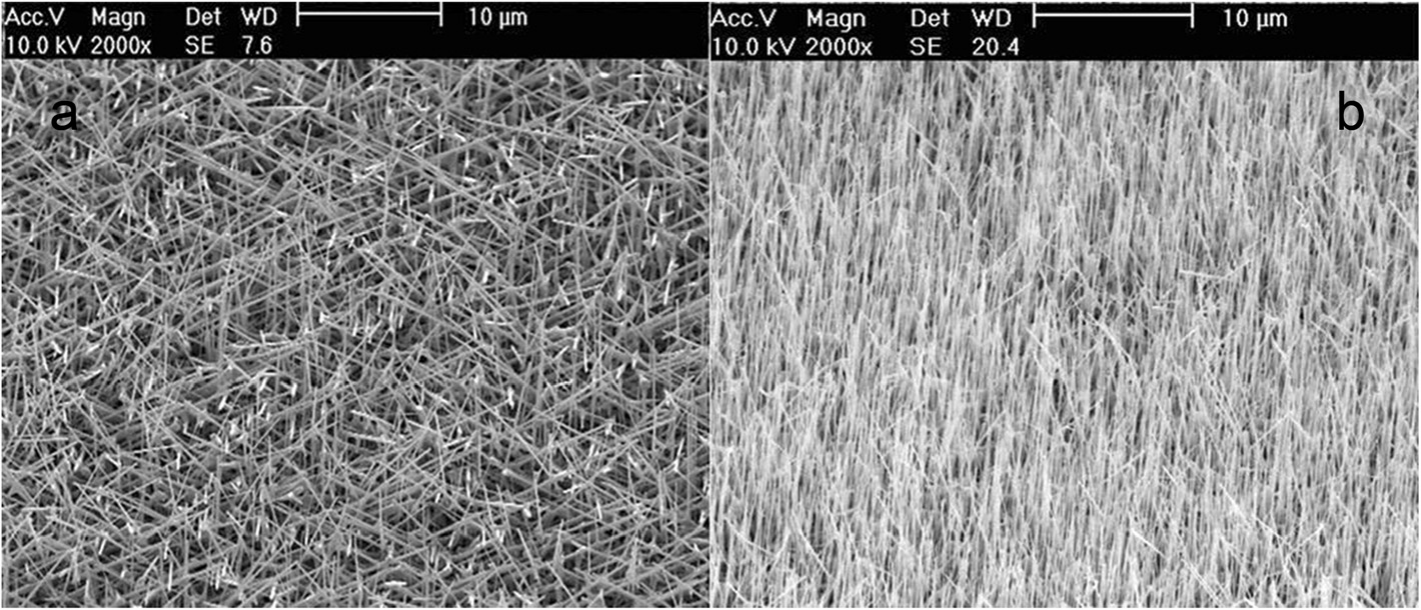
SEM images of as-grown GaAs/AlGaAs CSNWs on **a** Si (100) and **b** Si (111) substrates. CSNWs are inclined on the (100) surface but are vertically oriented on the (111) surface

Tilted view SEM micrographs of GaAs/AlGaAs CSNWs grown on Si (100) and Si (111) are shown in Fig. 1a,b, respectively. The CSNWs have an average lateral size of 125 nm. Most of the CSNWs on Si (100) are believed to have grown in the four equivalent <111> directions, inclined from the substrate surface, and distributed 90° apart azimuthally. This is a consequence of the preferential growth of NWs in the <111> direction [8, 15]. Hence, CSNWs on Si (111) have a growth direction that is predominantly perpendicular to the substrate surface. The NW density on both samples is approximately 10^8^ NWs per square centimeter. In Au-assisted VLS growth of GaAs NWs, growth area is dictated by the lateral size of liquid Au-Ga alloy droplet and the diameter of NWs is approximately given by the size of Au nanoparticles [16, 17]. Consequently, the two CSNW samples are expected to have similar GaAs core diameters since a single nanoparticle array template was fabricated on both substrates. Using the uncoated GaAs NW sample, the estimated core diameter is about 75 nm.

**Fig. 2:**
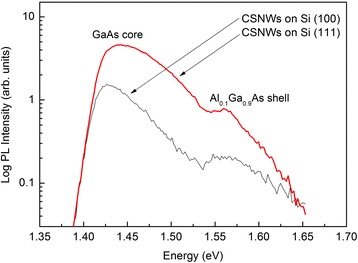
RT-PL spectra of GaAs/AlGaAs core-shell nanowires on Si (100) and Si (111) substrates. The two samples showed bulk-like emission peaks close to GaAs and Al_0.1_Ga_0.9_As bandgaps. GaAs PL emission intensity of the sample grown on Si (111) is roughly three times higher than that of the sample on Si (100). The PL intensity of Al_0.1_Ga_0.9_As relative to GaAs is 30 % better when CSNWs are grown on Si (111) compared to Si (100)

The RT-PL spectra of CSNWs on Si (111) and Si (100) are shown in Fig. 2, indicating bulk-like GaAs and Al_0.1_Ga_0.9_As bandgap emission peaks. Emissions with energies at 1.427 and 1.558 eV are attributed to GaAs and Al_0.1_Ga_0.9_As sections, respectively, of the CSNWs on Si (100). The emissions at 1.434 and 1.560 eV correspond to the GaAs and Al_0.1_Ga_0.9_As components of the CSNWs on Si (111). The well-defined PL spectra from the two samples at 300 K indicate that both GaAs and AlGaAs components of CSNWs have good optical and crystalline quality.

However, GaAs PL emission intensity of the sample grown on Si (111) is roughly three times higher than that of the sample on Si (100). This can be explained by better AlGaAs shell passivation on Si (111). When the NWs are perpendicular to the substrate surface and the substrate is rotated during growth, AlGaAs deposition on the surfaces of GaAs NWs is uniform [18]. On the other hand, the inclined orientation of NWs from a substrate surface produces partial AlGaAs passivation due to selective deposition of well-directed molecular beams only on the exposed NW facet [18]. Surface states in some parts of NWs without passivation allow photoexcited carriers to recombine nonradiatively, thus decreasing the PL intensity [19]. Based on Fig. 2, the PL intensity of Al_0.1_Ga_0.9_As relative to GaAs is 30 % better when CSNWs are grown on Si (111) compared to Si (100). This is consistent with the idea of uniform Al_0.1_Ga_0.9_As shell on Si (111)-grown CSNWs.

**Fig. 3:**
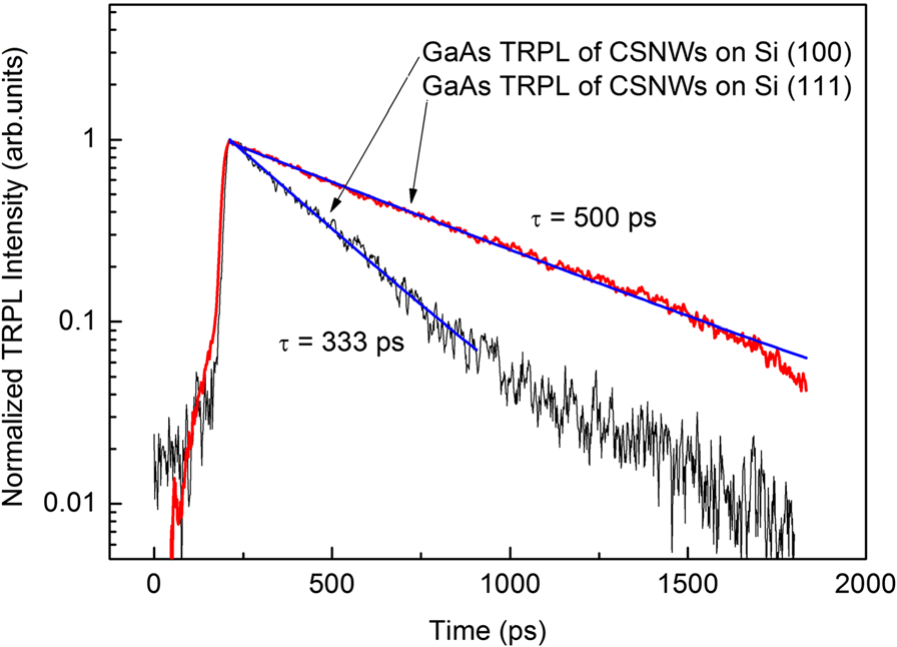
GaAs core TRPL of core-shell nanowires on Si (100) and Si (111) at room temperature. The carrier recombination rate of the GaAs core on Si (100)-grown CSNWs is faster than that on Si (111)-grown sample

The samples were also characterized by TRPL spectroscopy at room temperature to analyze carrier dynamics within the GaAs core of CSNWs grown on Si (100) and Si (111). Generation of carriers is manifested as the TRPL rise while recombination of carriers is described by the TRPL decay in Fig. 3. The rise time constant is fixed at 10 ps, which is also the resolution of our TRPL setup. Although the GaAs TRPL data of Si (100)-grown CSNWs seems to be not entirely monoexponential, two time constants cannot be fitted with confidence in the said curve since the TRPL intensities from 808 up to 1798 ps have very low signal-to-noise ratio (SNR). This feature is a consequence of the fact that GaAs PL emission intensity of the sample grown on Si (100) is less than that of the sample on Si (111). On Si (111)-grown CSNW sample, the TRPL of GaAs core is characterized by a single exponential decay rate. Hence, it is more appropriate to use decay time fitting that has a single rate on both samples. GaAs core’s carrier recombination lifetime, τ, in CSNWs grown on Si (100) is shorter than that on Si (111) as shown in Fig. 3. Since the two samples were grown side-by-side in an MBE chamber and had the same growth direction, crystalline quality and density of defects on GaAs core of the CSNWs are presumably identical. Thus, the longer recombination lifetime of carriers within the GaAs core of CSNWs grown on Si (111) is due to superior AlGaAs passivation of surface states [19].

**Fig. 4:**
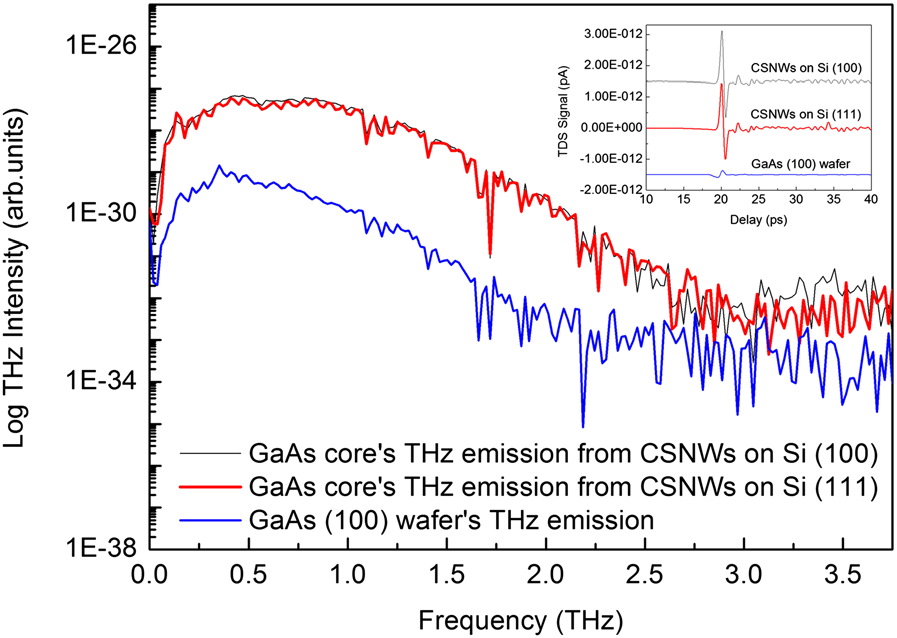
Logarithmic THz intensity of the GaAs core and semi-insulating GaAs (100) substrate. THz emission from the GaAs component of CSNW samples is one order of magnitude higher than that of GaAs wafer. The inset shows the corresponding time domain data, indicating that the generation of THz takes place in less than 5 ps. The signals were offset vertically for clarity

THz-TDS measurements were performed to shed light on the drift of photoexcited carriers by the surface depletion field. The logarithmic THz emission spectra and the corresponding time domain data (inset) generated from ultrafast photoexcitation of GaAs core and SI-GaAs (100) substrate are shown in Fig. 4. Generation of THz took place in less than 5 ps based on the time domain data. THz emission from the GaAs component of CSNW samples was one order of magnitude higher than that of GaAs wafer. The morphology of the grown CSNWs, as dictated by the Si substrate orientation, does not affect their THz emission efficiency. Results indicate a negligible difference on the intensity and bandwidth of THz radiation emitted from the two samples. This supports the fact that the deposited GaAs material on both substrates produced samples with comparable NW densities and similar GaAs core diameters. The samples’ difference in GaAs core’s carrier recombination lifetime did not influence THz emission since the two processes involve distinct mechanisms.

**Fig. 5:**
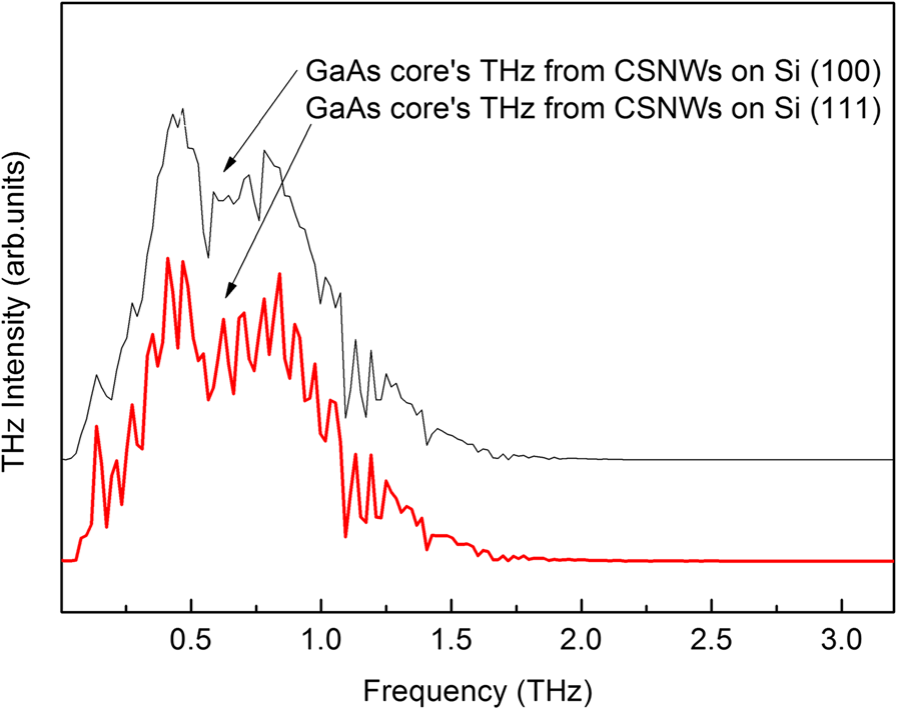
THz intensity of the GaAs component of CSNWs on Si (100) and Si (111). Fabry-Perot modes are visible in the Si (111)-grown sample. The spectra were offset vertically for ease of comparison

The THz emission intensity profile shown in Fig. 5 reveals Fabry-Perot modes only on the spectrum of CSNWs grown on Si (111). The separation between any two adjacent Fabry-Perot peaks, Δ*f*, in the graph matches with an optical cavity length *L* that can be calculated using the equation:

1 $$ \Delta f=\frac{c}{2Ln \cos \theta } $$

where *c* is the speed of light and *n* is the index of refraction [20]. The angle of incidence *θ* is equal to 45° because of the reflection excitation geometry setting of the THz-TDS setup. Using *n* = 3.42 for the index of refraction of Si at THz frequencies [21, 22], the cavity length is estimated to be 820 μm. It is interesting to note that the calculated value for *L* is in the same order of magnitude as the thickness of the Si (111) substrate. The oscillatory modes that were observed on the Si (111)-grown sample are attributed to highly directional THz beam propagation, as dictated by the drift of carriers along the NW length. For vertical NWs, such as the Si (111)-grown sample shown in Fig. 1, the THz dipole radiation pattern is parallel to the substrate surface [23, 24]. This geometry ensures THz directionality as it undergoes multiple reflections within the substrate. It has been observed that reflections at the internal interfaces of a Si substrate can manifest as Fabry-Perot modes in the THz intensity profile, when THz waves interact with the planar material [22].

In summary, TRPL alongside with THz emission measurements enabled us to look at carrier recombination dynamics vis-à-vis carrier transport at room temperature. Carrier dynamics in two different temporal regimes were studied. In the sub-nanosecond time scale (300–500 ps), time-resolved radiative recombination efficiency of carriers was examined. In the 0–4 ps range, surface field-driven ballistic transport of carriers was probed in terms of the radiated THz waves. The samples’ difference in GaAs core’s carrier recombination lifetime did not influence THz emission since the two processes involve distinct mechanisms.

## Conclusions

GaAs/Al_0.1_Ga_0.9_As CSNWs were grown using AAO-patterned gold nanoparticles on Si (100) and Si (111) substrates. The nanowire density on both samples is around 10^8^ NWs/cm^2^. RT-PL from the samples showed bulk-like emission peaks close to GaAs and Al_0.1_Ga_0.9_As bandgaps. The Si (111)-grown CSNWs produced an enhanced luminescence due to uniform Al_0.1_Ga_0.9_As shell passivation. TRPL measurements at 300 K obtained shorter GaAs carrier recombination lifetime for CSNWs grown on Si (100) than that on Si (111) because of better passivation of surface states on the GaAs core of CSNWs grown on the latter. Ultrafast optical excitation of GaAs core on the two samples generated a negligible difference in the intensity and bandwidth of emitted THz radiation since the deposited GaAs material on both substrates formed samples with comparable NW densities and similar GaAs core diameters. The THz spectrum of CSNWs grown on Si (111) exhibited Fabry-Perot modes that originated from the multiple reflections of THz waves within the Si substrate. Our results on the carrier dynamics of Si (100) and Si (111) substrate-grown GaAs/AlGaAs CSNWs are believed to be valuable in designing functional NW-based photovoltaic and optoelectronic devices.

## Abbreviations

AlGaAs: aluminum gallium arsenide
GaAs: gallium arsenide
Si: silicon
